# Emergy evaluation of ecological sustainability in Cananguchales (*Mauritia flexuosa*): A biocountable approach in the Amazonian foothills

**DOI:** 10.1371/journal.pone.0358076

**Published:** 2026-09-25

**Authors:** Víctor Julio Balanta Martínez, Oscar Fabian Patiño Perdomo, Faver Álvarez

**Affiliations:** 1 University of the Amazonia, Faculty of Accounting, Economic and Administrative Sciences, Florencia, Caquetá, Colombia; 2 University of the Amazonia, Faculty of Engineering, Florencia, Caquetá, Colombia; 3 University of the Amazonia, Faculty of Agricultural Sciences, Florencia, Caquetá, Colombia; King Mongkut’s University of Technology North Bangkok, THAILAND

## Abstract

Cananguchales, forested wetlands dominated by *Mauritia flexuosa*, are strategic ecosystems in the Amazonian foothills due to their contribution to ecosystem goods and services. However, their high vulnerability to climate variability and the progressive degradation caused by the global crisis demand new tools for their valuation and sustainable management. This study evaluated the ecological sustainability of Cananguchales through emergy indicators that allow biocountable monitoring of the efficiency and performance of their ecological wealth. Indicators of Ecological Loading, Sustainable Emergy, Investment, Yield, and Renewability were applied within a non-experimental design using a quantitative, applied, and deductive–projective approach. Using ecological data and energy flow equations, values were estimated in joules, later transformed into solar emergy joules and emergy-based US dollars using standardized factors. The results showed a high proportion of renewable energy inputs (>99%) over five consecutive years, demonstrating high energy efficiency and ecological stability. A unified biocountable structure was established for these wetlands. The emergy indicators applied here are established tools; the contribution of the study lies at the level of accounting recognition, namely the integration of emergy synthesis into a formal double-entry biocountable structure in which ecological flows are recognised as debit assets and credit liabilities and closed into an ecological equity balance. It is concluded that this accounting-recognition structure provides an operational route for objectively valuing strategic ecosystems and guiding decisions towards their conservation and sustainability in the Amazonian context.

## Introduction

Cananguchales are perennial woody formations of *Mauritia flexuosa*, a species of the Arecaceae family endemic to the tropical forests of South America in the Amazon basin [1]. This aquatic ecosystem is considered strategic due to its provision of ecosystem services such as sediment filtration from water, carbon sequestration, and flood control, among others [2]. The value of this ecosystem lies in its essential role in water regulation, carbon storage, and biodiversity conservation—critical factors for the ecological integrity and survival of the Amazon region. Consequently, the ongoing degradation of Cananguchales caused by anthropogenic activities—such as extensive cattle ranching and deforestation—calls for the use of accounting tools in ecosystem valuation, due to the importance of these systems in providing ecosystem services.

The incorporation of biophysical valuation methods such as emergy analysis enables an objective estimation of ecological assets and their dynamics of generation and use. This valuation facilitates the construction of biocounting systems that make visible the existence, appropriation, and circulation of natural wealth, thereby strengthening decision-making processes aimed at conservation and sustainable use. Mejía [3], Álvarez [4], and Brown et al. [5] emphasize the need to move toward ecological accounting that explicitly acknowledges environmental goods and services, contributing to the safeguarding of future generations’ rights. This approach aligns with critical and heterodox schools of accounting that frame it as a social, political, and moral science, part of an autopoietic system composed of the biotic and abiotic components of the biosphere—considered as a living and incorruptible unit [6].

Thus, sustainability is defined as an integrative and ecocentric approach to human and territorial development, based on recognizing the biophysical limits of ecosystems and the need to respect their carrying capacity and resilience [6–10]. Unlike anthropocentric conceptions of sustainable development, sustainability entails a paradigmatic shift that prioritizes intergenerational equity, social justice, and respect for life in all its forms [11–14]. According to Guimarães [15], it implies a new ethic of development linked to bioethics, while Wilkinson et al. [16] consider it a tool for rational planning from an ecocentric perspective. Measuring sustainability requires methodologies that integrate biophysical, social, and economic dimensions—going beyond traditional approaches focused on financial data and indicators [5,15,17–19].

In this regard, biocounting is defined as an accounting information system oriented toward sustainability. It integrates both qualitative and quantitative valuation of the existence and circulation of ecological, social, and economic wealth managed by organizations, within a unified and common structure [3,4,6,20,21]. In contrast to traditional anthropocentric accounting, biocounting incorporates biophysical valuation methods such as emergy analysis, which enable the recording of energy, matter, and information flows in socio-ecological systems [22–28].

Smith et al. [14] and Kagan [10] argue that accounting systems must integrate not only price-related elements but also biotic and abiotic components in the representation of natural wealth. This integration enables rational resource management and decision-making based on strong sustainability, advocating intergenerational equity. Consequently, biocounting systems—by incorporating the laws of thermodynamics into ecological wealth valuation—allow the articulation of variables and sustainability indices for the objective evaluation of strategic ecosystems, addressing the need to integrate ecological values into the accounting language.

Some explorations, such as emergy accounting, propose a fundamental tool for evaluating ecological and productive systems. Odum [29] introduced the basic principles, suggesting that the energy used directly and indirectly to produce a product can be quantified through emergy. Odum [30] expanded the discussion by establishing an energy hierarchy based on biogeochemical cycles, while Brown [29] introduced the concept of “formation” to separate emergy associated with materials, energy, and information, thereby improving model accuracy. Likewise, Brown & Herendeen [31], Tilley & Brown [32], Felix & Tilley [20], Felix [33], Brown et al. [5], and Zhang et al. [22] have deepened emergy analysis through assessments of various complex systems and optimization of sustainability-based management. Meanwhile, Collins [13], Giannantoni [34,35], and Martin [36] have contributed to the theoretical development of transformity logic and emergy valuation in ecosystems.

This method allows for the objective valuation of ecological wealth generation and use processes by incorporating indicators such as emergy yield ratio, ecological loading ratio, and renewability [17,37]. According to López & Rodríguez [38], emergy analysis is a methodological alternative for evaluating the impacts of human activities on ecosystems and helps formulate sustainable management policies [5,22,25,37,39–42].

Therefore, monitoring ecological wealth involves systemic supervision or tracking of the flows of energy, matter, and information circulating within a strategic ecosystem, which enables assertive decision-making for its conservation, restoration, protection, and safeguarding [3,4]. This control is based on biophysical accounting valuations, such as emergy synthesis, which makes it possible to measure changes in the availability and quality of ecosystem wealth while ensuring that the carrying capacity and resilience of ecosystems are respected—fundamental elements in achieving intergenerational sustainability [5,10,14,29]. Similarly, Ceballos & Mejía [20] and Pulselli et al. [25,26] argue that effectiveness must be evaluated through biophysical indicators that reflect the persistence of ecological functionality under anthropogenic pressure, which in turn supports the development of timely strategies and interventions to ensure sustainability.

At the same time, the performance of ecological wealth emphasizes the efficiency with which an ecosystem transforms available resources into ecosystem goods and services [29,43] Thus, performance is assessed using indices that evaluate whether an ecosystem is operating optimally relative to its natural capacity [39,42]. Consequently, performance relates to the ability of an ecosystem to amplify the generation of ecological wealth with minimal external inputs—a key indicator for sustainability-oriented decision-making [17,38].

## Materials and methods

This research aimed to evaluate the sustainability of Cananguchales (*Mauritia flexuosa*) through emergy flows, contributing to the biocountable control of the efficiency and performance of ecological wealth based on the Ecological Loading Ratio (ELR), Sustainable Emergy Index (ESI), Emergy Investment Ratio (EIR), Emergy Yield Ratio (EYR), and Renewability Index (%R). The methodological approach was based on an applied research nature, with a deductive–projective logic and a non-experimental design with a quantitative approach. It employed an exploratory scope and a non-probabilistic observation method, selecting a Cananguchal that met the research criteria as the unit of analysis [44].

This study adopts an intensive analytical approach; therefore, the results should be interpreted as functional representations of the system rather than as statistically generalizable inferences.

This research is framed within multidimensional accounting theory and is therefore not a basic-science study of ecosystem ecology. Emergy synthesis is adopted here as a measurement method that makes it possible to recognise, measure and disclose ecological wealth within an accounting information system; the ecosystem is treated as a closed, non-intervened system precisely because such conditions isolate the recognition and measurement problem from the effects of anthropogenic intervention. Accordingly, the object of study is the accounting representation of ecological wealth — its recognition as assets and liabilities, its measurement in a common unit, and its disclosure in a structured statement — rather than the ecological modelling of the wetland itself. The unit of analysis therefore functions as a demonstration case for a recognition, measurement and disclosure structure that is intended to be applicable to any type of organisation and to any type of wealth (ecological, social or economic), within the construction of an integral accounting system [3,21,45].

### Research Phases

**Fig 1:**
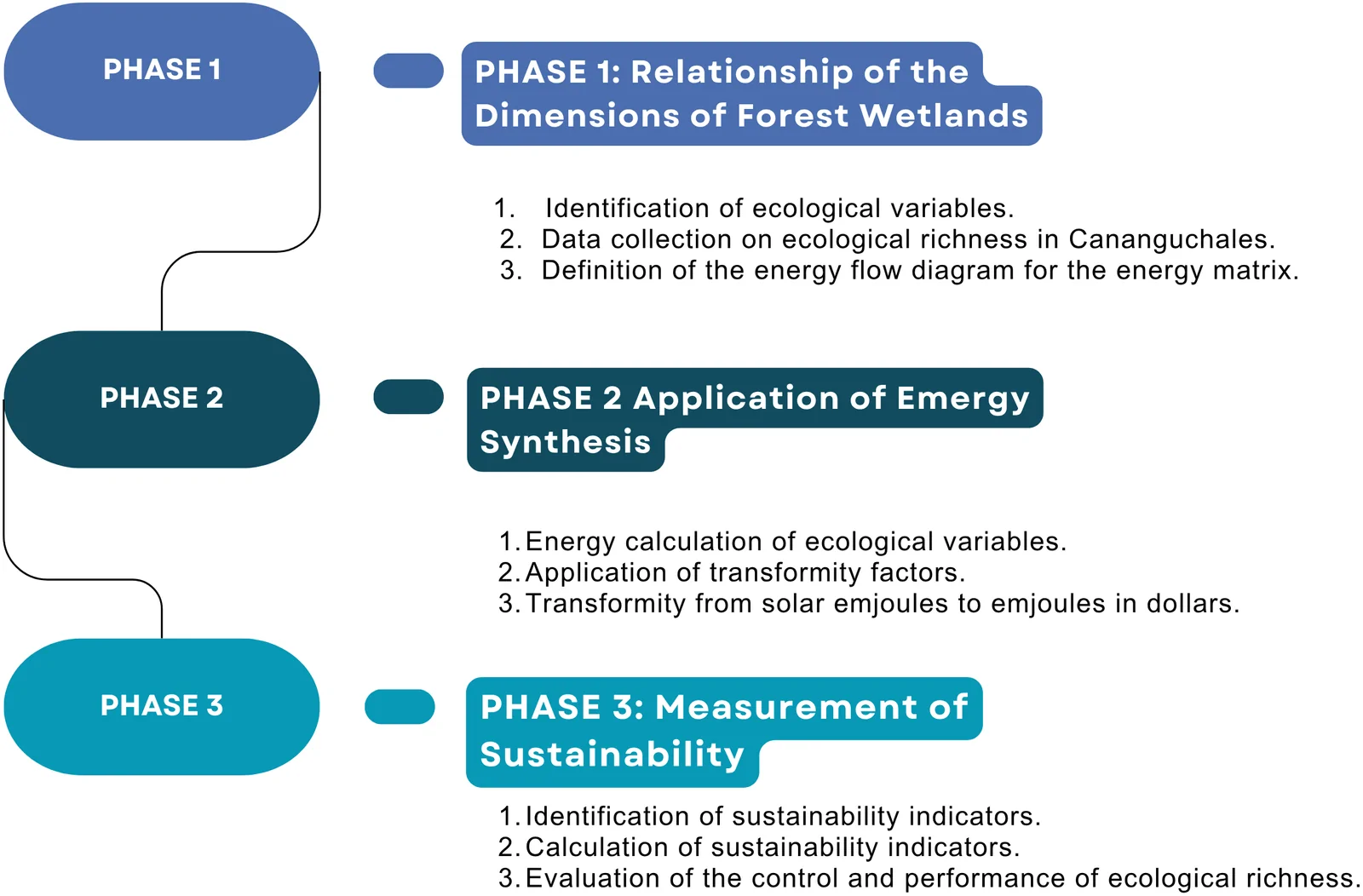
General aspects of the applied methodological pathway. *Note*. The figure shows the general aspects of the methodological route applied to support the emergy analysis as a valuation method for the biocounting of ecological wealth in wetlands dominated by *Mauritia flexuosa*. Source: Own elaboration (2025).

Fig 1 illustrates the research phases according to the proposed methodology. Initially, an ecological foundation was established by identifying key variables for the study, collecting biodiversity data in specific sites such as Cananguchales, and developing an energy flow diagram to contextualize ecosystem dynamics. Subsequently, emergy synthesis was applied as a quantitative approach that translates ecological interactions into energetic and economic terms using transformity to convert solar emergy joules into monetary units. This sequential process ensured the integration of biophysical and economic criteria, which are essential for understanding the true energetic value of the ecosystem services provided by Cananguchales. Finally, sustainability was measured using the analyzed indices, allowing for the evaluation of control and performance over ecological wealth.

### Unit of Analysis

The unit of analysis for the study was the Cananguchales located within the Macagual Amazon Research Center “César Augusto Estrada González” of the University of the Amazon. This area is situated 22 km from the urban center in a rural zone, covering approximately 383.36 ha^−1^, comprising pastures, forest germplasm, and wetlands dominated by *Mauritia flexuosa* palms (Universidad. For data collection, a non-directed sampling method was used to determine the ecological valuation units (EVUs), which were selected based on the following criteria:

- Wetlands with perennial woody vegetation with a minimum 50% cover of Cananguchales or *Mauritia flexuosa*.
- Cananguchales located in a foothill landscape.
- Area ranging from 0,5 ha^−1^ y 5 ha^−1^.
- Located within the Macagual Research Center territory.

Ecological Valuation Units (EVUs) were defined as spatially delimited ecological systems exhibiting internal functional coherence in terms of energy flows, hydrological dynamics, and biotic structure, enabling their analysis as integrated valuation units. Their delimitation was based on the identification of areas with a predominance of *Mauritia flexuosa* and homogeneous ecological conditions, considering both the continuity of ecosystem processes and the stability of their biophysical components. In this sense, EVUs were not conceived as statistical units, but rather as functional units that allow the reconstruction and representation of the system’s ecological metabolism. The final selection was based on criteria of ecological integrity, availability of continuous environmental information, and absence of significant anthropogenic intervention, which ensured consistency in the measurement of variables and their subsequent integration into emergy synthesis. Likewise, the internal heterogeneity of each unit was considered through the observation of structural and functional patterns of the ecosystem, ensuring that the values obtained corresponded to representative conditions of Cananguchal functioning within the Amazonian foothill context.

Two (2) perennial woody wetlands classified as Cananguchales, due to their high density of *Mauritia flexuosa*, were selected as units of analysis, encompassing a total undisturbed area of 1.78 hectares, it is worth noting that, due to the presence of a meteorological station in the area, data were available from 2017 to 2021.

### Identification of Prioritized Variables

**Fig 2:**
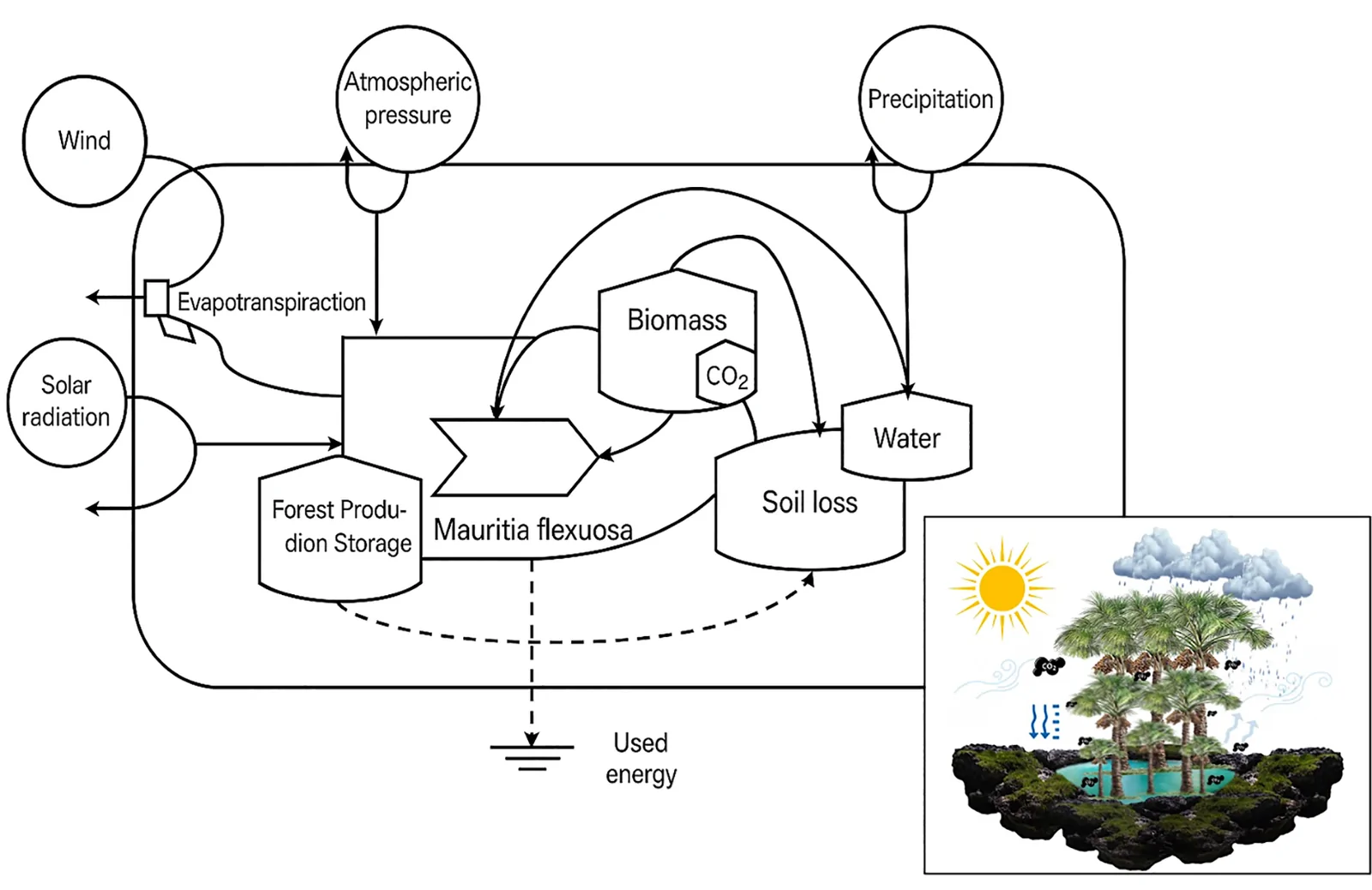
Undisturbed Cananguchales (*Mauritia flexuosa*) ecosystem. Note. The figure shows the ecosystem dynamics of undisturbed Cananguchales (*Mauritia flexuosa*) forested wetlands, which served as the unit of analysis. Source: Own elaboration (2025).

To prioritize the most material variables related to the provisioning ecosystem services of non-anthropogenically disturbed Cananguchales in the Amazonian foothills (Fig 2), emergy synthesis was conducted. First, the ecological assets and liabilities were quantified in joules (J) using the relevant formulas (Table 1). These results were then converted into solar emergy joules (Sej) using a transformation factor derived from a literature review of recent emergy studies. The final step involved converting Sej into emergy-based US dollars using the Gross Domestic Product (GDP) data from the Caquetá region for each year. This process allowed for establishing a common unit of valuation and assessment of ecological components within the biocounting framework.

**Table 1:** General aspects of the methodological approach applied.

Variable	Dynamic	Unit	Formula	Reference
a. Solar radiation	E	j. year^−1^	$E_{sol}=n\left(\frac{Wh}{m^{\text{2}}dia}\right)3.600\;t.$	Odum [24]
b. Wind energy	E	j. year^−1^	$E_{V}=F_{V}V_{V}t$.	Vivas & Brown [46]
c. Rainfall	E	j. year^−1^	$E_{L}=LA\rho E_{g}(\text{1}-\varepsilon )$	Odum [30]
d. Atmospheric pressure	E	j. year^−1^	$E_{P}=\left(\frac{P}{h^{\prime }}\right)At$.	Vivas & Brown [46]
e. blue water	P	l. year^−1^	$E_{AA}=(A*P)*1000$	Rodríguez [47]
f. carbon storage	P	kg. year^−1^	$EC{𝐨}_{2}=(A*\;𝐧𝐩)*𝐂{𝐎}_{2}$	Rodríguez [47]
g. storage forest production	P	j. year^−1^	$EPS=𝐀*𝐩𝐬*𝐌𝐨*\left(1-𝐌𝐨{𝐇}_{2}𝐎\right)*𝐄𝐦𝐨*𝐅𝐂$	Prado-Jatar & Brown [48]
h. Evapotranspiration	P	j. year^−1^	$E_{ev}=(\frac{ev}{R})At$.	Aguilar et al. [7]
i. Remaining energy	P	j. year^−1^	*ER = E. entrada – E. almac – E. de salida*	Residual balance
j. Soil loss	S	j. year^−1^	$E_{ps}=(ps)M_{O}E_{MO}$.	Vivas & Brown [46]
k. Conservation of Energy			$\sum_{i=\text{1}}^{n}E_{i}=\text{0}$.	Balance
**Nomenclature a**				
*n* = Amount of kWh per m²·day. *Wh.m*^*2*^*.día* = Watts per square meter per day. *3.600* = Constant corresponding to the number of joules of energy produced by 1 Wh. *t* = Number of days of exposure.				
**Nomenclature b**				
*F*_*v*_ = Wind force acting on the Cananguchal. *V*_*v*_ = Wind velocity measured in meters per second. *t* = Time measured in seconds.				
**Nomenclature c**				
*L* = Precipitation associated with rainfall, expressed as a multiannual monthly average, measured in mm. *A =* Area, corresponding to one hectare or 10,000 m². *ρ* = Density of pure water, with a value of 1000 kg·m^−^³. *Eɡ* = Gibbs free energy, generically represented as 237.2 (kJ·mol^−^¹) = 237.2 × (1,000 J/ 18.015 g). Ɛ = Runoff.				
**Nomenclature d**				
*P* = Atmospheric pressure at the elevation of the Cananguchal. *ℎ´* = Elevation of the Cananguchal above sea level. *A* = Area of the stand, equivalent to 1 ha. *t* = Time in seconds.				
**Nomenclature e**				
*A* = Area of the Cananguchal. *P* = Depth. *1000* = Constant corresponding to the conversion of 1 m³ to liters (L).				
**Nomenclature f**				
*A* = Area of the Cananguchal, expressed in hectares (ha). *np* = Number of palms per hectare. *CO*_*2*_ = Carbon stock of the Cananguchal area.				
**Nomenclature g**				
*Ea* = Value of the energy stored by the ecosystem. *Ec* = Value of the energy stored in joules per culm of the Cananguchal. *c* = Total number of culms (Cananguchal units) per hectare. *Pc* = Average weight in kilograms of each culm (Cananguchal unit) per hectare. *t*_*ma*_ = Time.				
**Nomenclature h**				
*ev* = Evapotranspiration measured at the plantation site. R = Standard radiation value, expressed in megajoules per m² per day (MJ·m^−^²·day^−^¹). *A* = Area of the Cananguchal. *t* = Time, expressed as the number of days per year.				
**Nomenclature i**				
E. input = Energy inputs. E.stored = Stored energy. E. output = Energy outputs.				
**Nomenclature j**				
*A* = Area of the Cananguchal. *ps* = Soil loss. *Mo* = Percentage of organic matter in the stand. *Mo*_*H2O*_ = Water content in organic matter. *E*_*mo*_ = Energy associated with organic matter. *FC* = Conversion factor in J/Kcal.				
**Nomenclature k**				
*∑* = Summation. *n* = Subscript indicating the energy value used. *i=1* = Subscript representing the unit index. *E*_*i* =_ All energy values of the ecosystem.				

*Note: The table shows the relationship between the dynamics, the unit of measurement, and the sample equations for the measurement variables of the Cananguchales (Mauritia flexuosa L.f.). Source: own elaboration (2025).*

In particular, carbon storage was treated through a sequential transformation process that ensured dimensional consistency between biophysical variables and their representation in energetic terms. Initially, this variable was estimated in mass units (kg·year^−^¹) based on plant biomass, using conversion factors widely reported in the literature. Subsequently, these values were converted into energy units (J) using energy coefficients associated with the calorific content of organic matter, and finally transformed into emergy units (sej) using the corresponding transformities. This procedure enabled the integration of carbon storage into the system of ecosystem energy flows, ensuring its comparability with the other variables considered in the emergy synthesis.

From this perspective, EVUs constitute the operational basis for the application of emergy synthesis, as they allow the integration, within a single analytical unit, of the flows of energy, matter, and information that define the functioning of the ecological system. This delimitation facilitates the identification of inputs (E), internal transformation processes ((P), and outputs or losses (S), ensuring consistency in the traceability of flows and their conversion into energy units (J) and subsequently into emergy (sej)). Consequently, the use of EVUs ensures that emergy-based valuation is not performed on isolated variables, but rather on functionally coherent systems, thereby strengthening the interpretation of sustainability indicators and their linkage to the ecological performance of the Cananguchal under natural conditions.

For the purposes of emergy synthesis, remaining energy was defined as a closing variable of the system’s energy balance, representing the fraction of energy that remains available within the system after accounting for the identified inputs, internal transformation processes, and ecological losses. Operationally, it was estimated as the difference between total system inputs and the sum of internal transformations and outputs, thereby approximating the energy retained or conserved within the functional structure of the ecosystem. This variable does not correspond to an independent flow, but rather to an integrated expression of the overall system behavior under an open-system perspective. Remaining Energy was calculated as the difference between total system inputs and the sum of internal transformations and outputs.

Table 1 presents the essential variables used to evaluate external energy inputs (E), internal production processes (P), and system losses (S), including solar radiation, wind energy, precipitation, and atmospheric pressure, all expressed in joules per year (J·year^−^¹). Internal production variables include blue water (liters per year), carbon storage (kilograms per year), forest production, and evapotranspiration, mostly expressed in energy terms (J·year^−^¹). System losses comprise remnant energy and soil loss, the latter estimated following the methodology by Vivas & Brown [46]. Within the emergy accounting framework, soil loss is recognised as an ecological liability rather than as a system input because it constitutes a non-renewable and uncompensated outflow: the pedogenetic emergy invested in forming that soil through geological and biological processes operating over very long time scales leaves the system faster than it can be regenerated. This treatment follows Odum’s [24] original classification of non-renewable resource losses, the wetland application of Vivas & Brown [46], and is consistent with the emergy-based sustainability assessments of Zhang et al. [49] and Bravo et al. [17], in which eroded soil is accounted for as a depletion of stored non-renewable emergy.

Ecological variables were measured using delineated ecological plots within each unit of analysis, taking into account the structure and composition of the Cananguchal under non-intervened conditions. To this end, field records associated with the biophysical characterization of the ecosystem were integrated with meteorological data series corresponding to the 2017–2021 period, obtained from the conventional and automatic meteorological station of the Institute of Hydrology, Meteorology and Environmental Studies (IDEAM) located at the Amazonian Research Center Macagual, which provides records of sunshine hours (pyranometer/heliograph), wind speed (anemometer), rainfall (pluviometer) and atmospheric pressure (barometer) at daily resolution, subsequently aggregated into annual series. Two complementary sources were therefore used: (i) primary field measurements collected in the delineated ecological plots of the two Ecological Valuation Units, corresponding to blue water, carbon storage, forest production and soil loss; and (ii) the 2017–2021 IDEAM meteorological series for the climatic variables. Carbon storage was estimated from plant biomass using conversion factors widely reported in the literature, while blue water was approximated based on the system’s water balance. Forest production was estimated as a function of biomass increment and vegetation accumulation dynamics, and soil loss was determined through approaches based on erosion models adapted to tropical conditions. This procedure ensured consistency between the measured ecological variables and their subsequent transformation into energy units (J), facilitating their integration into the emergy synthesis. Furthermore, in order to reduce the uncertainty inherent in the estimation of biophysical variables, reference coefficients reported in previous studies were employed and cross-validated against regional ecological parameters.

## Results and analysis of the research

Emergy analysis can be considered a biocountable method for estimating the energy flows of ecological wealth within a system under a common unit. After calculating the prioritized variables of provisioning ecosystem services from wetlands dominated by *Mauritia flexuosa* in joules (J), each was multiplied by its respective transformity factor to convert it into solar emergy units (Sej), and subsequently into emergy-based US dollars (US$), as shown in Table 2.

**Table 2:** Emergy synthesis of the provisioning ecosystem services of wetlands with Canangucha (*Mauritia flexuosa* L.f.) for biocountable processes corresponding to each year.

Emergy Flow	Unit	Flow Value (Unit/year)					Transformity (Sej/unit)	Source of Transformity	Emergy (Sej/year)				
		2017	2018	2019	2020	2021			2017	2018	2019	2020	2021
**Renewable Resources**													
Solar Radiation	J	7.76E + 13	7.23E + 13	5.92E + 13	6.97E + 13	8.17E + 13	1.00E + 00	Odum [24],	7.76E + 13	7.23E + 13	5.92E + 13	6.97E + 13	8.17E + 13
Wind Energy	J	8.16E + 10	8.26E + 10	8.58E + 10	8.67E + 10	9.32E + 10	2.45E + 03	Vivas & Brown [46],	2.00E + 14	2.02E + 14	2.10E + 14	2.12E + 14	2.28E + 14
Rainfall	J	1.92E + 15	2.01E + 15	1.86E + 15	1.45E + 15	1.94E + 15	3.05E + 04	Odum [30],	5.85E + 19	6.14E + 19	5.66E + 19	4.44E + 19	5.93E + 19
Atmospheric Pressure	J	4.18E + 25	2.24E + 25	2.38E + 25	2.74E + 25	3.82E + 25	2.45E + 03	Vivas & Brown [46],	1.02E + 29	5.50E + 28	5.84E + 28	6.70E + 28	9.36E + 28
Blue Water	J	1.90E + 18	1.67E + 18	1.61E + 18	1.66E + 18	2.06E + 18	2.37E + 11	Rodríguez [47],	4.50E + 29	3.97E + 29	3.81E + 29	3.92E + 29	4.88E + 29
Carbon Storage	Kg	6.65E-01	5.87E-01	5.64E-01	5.81E-01	7.23E-01	4.76E + 14	Rodríguez [47],	3.17E + 14	2.79E + 14	2.68E + 14	2.76E + 14	3.44E + 14
Forest Production Storage	J	1.04E + 09	9.20E + 08	8.84E + 08	9.10E + 08	1.13E + 09	3.49E + 04	Prado-Jatar & Brown [48],	3.64E + 13	3.21E + 13	3.08E + 13	3.18E + 13	3.95E + 13
**Non-Renewable Resources**													
Evapotranspiration	J	2.11E + 08	1.86E + 08	1.79E + 08	1.84E + 08	2.30E + 08	2.59E + 04	Aguilar et al [7],	5.47E + 12	4.83E + 12	4.64E + 12	4.78E + 12	5.94E + 12
Soil Loss	J	5.71E + 08	5.03E + 08	4.83E + 08	4.98E + 08	6.20E + 08	7.40E + 04	Vivas & Brown [46],	4.22E + 13	3.73E + 13	3.58E + 13	3.68E + 13	4.59E + 13
**Remaining Energy**		4.18E + 25	2.24E + 25	2.38E + 25	2.74E + 25	3.82E + 25	4.76E + 14	By definition (residual balance)	5.52E + 29	4.52E + 29	4.39E + 29	4.59E + 29	5.82E + 29
**Energy Conservation (E)**		0.00E + 00	0.00E + 00	0.00E + 00	0.00E + 00	0.00E + 00	0.00E + 00	By definition (residual balance)	0.00E + 00	0.00E + 00	0.00E + 00	0.00E + 00	0.00E + 00

*Note: The table shows the biocentric valuation represented in the synthesis of the most material variables of the undisturbed Cananguchales (Mauritia flexuosa) in the Amazonian foothills. Units are in Joules (J) and Kilograms (Kg), in addition to the sources of the transformations.* Remaining Energy and Energy Conservation are not independent ecosystem flows, but closing (balance) variables of the system’s energy accounting, computed as ER = ΣE inputs – ΣE stored – ΣE outputs (Table 1, nomenclature i). Because atmospheric pressure is several orders of magnitude larger than any other input, it numerically dominates the input term; consequently the residual value of Remaining Energy is, to three significant figures, very close to the atmospheric-pressure flow value. This proximity is an expected outcome of the balance equation and does not represent a duplicated entry or a calculation error. Likewise, the transformity assigned to Remaining Energy (4.76E + 14 sej/unit) is an implicit value derived from the closing balance itself (“by definition”) and is not copied from the carbon-storage transformity; both appear identical only because they are rounded to three significant figures for presentation. All remaining rows were verified against the source spreadsheet, where emergy equals the flow value multiplied by its transformity (e.g., Blue Water 2017: 1.90E + 18 L × 2.37E + 11 sej/L = 4.50E + 29 sej; Carbon Storage 2017: 0.665 kg × 4.76E + 14 sej/kg = 3.17E + 14 sej; Atmospheric Pressure 2017: 4.18E + 25 J × 2.45E + 03 sej/J = 1.02E + 29 sej). *Source: own elaboration (2025).*

In this context, the table presents the flows of renewable and non-renewable energy from 2017 to 2021, incorporating the annual values for each flow, their respective transformities, and bibliographic sources. The data reveal a diversity of energy sources, with renewable resources such as solar radiation, wind energy, precipitation, and atmospheric pressure accounting for the majority of available energy. In contrast, non-renewable resources—such as evapotranspiration and soil loss—show significantly lower energy volumes, albeit with higher transformity values, indicating a greater amount of energy required per unit. Notably, the transformity values of certain flows, such as carbon storage and blue water, reach magnitudes in the range of 10¹¹–10¹⁴ sej/unit, suggesting a high prior energy investment required for their formation or conservation.

Throughout the five-year period analyzed, solar radiation and atmospheric pressure flows maintained considerable magnitudes, albeit with interannual variations attributable to climatic conditions or specific regional measurements. Likewise, a slight upward trend was observed in flows such as wind energy and carbon storage in 2021, possibly linked to changes in ecological dynamics or environmental restoration processes. The inclusion of remaining energy made it possible to interpret system behavior beyond direct input and output flows, as it represents the portion of energy that remains available within the functional structure of the ecosystem after transformation processes and identified losses. From this perspective, remaining energy is understood as an expression of the residual ecological balance of the Cananguchal, reflecting its capacity for energy conservation and retention within an open system. Its behavior throughout the analyzed period provides insights into the functional stability of the wetland and its relationship with ecological sustainability as assessed through emergy-based indicators.

The emergy flow diagrams illustrate the systemic interaction between renewable and non-renewable energy flows that influence the ecological functioning of *Mauritia flexuosa* over five consecutive years. A functionally stable structure is observed, where components such as solar radiation, wind, precipitation, and atmospheric pressure act as the system’s primary inputs, supporting key processes like evapotranspiration, carbon storage, forest production, and biomass generation. Biomass, in turn, feeds back into the system through CO_2_ capture, while water and soil loss represent relevant output flows linked to hydrological and soil degradation processes. This systemic dynamic reveals the complexity of energy exchanges within the analyzed ecosystem and underscores the central role of *Mauritia flexuosa* as a key organizer of the local ecological metabolism

**Fig 3:**
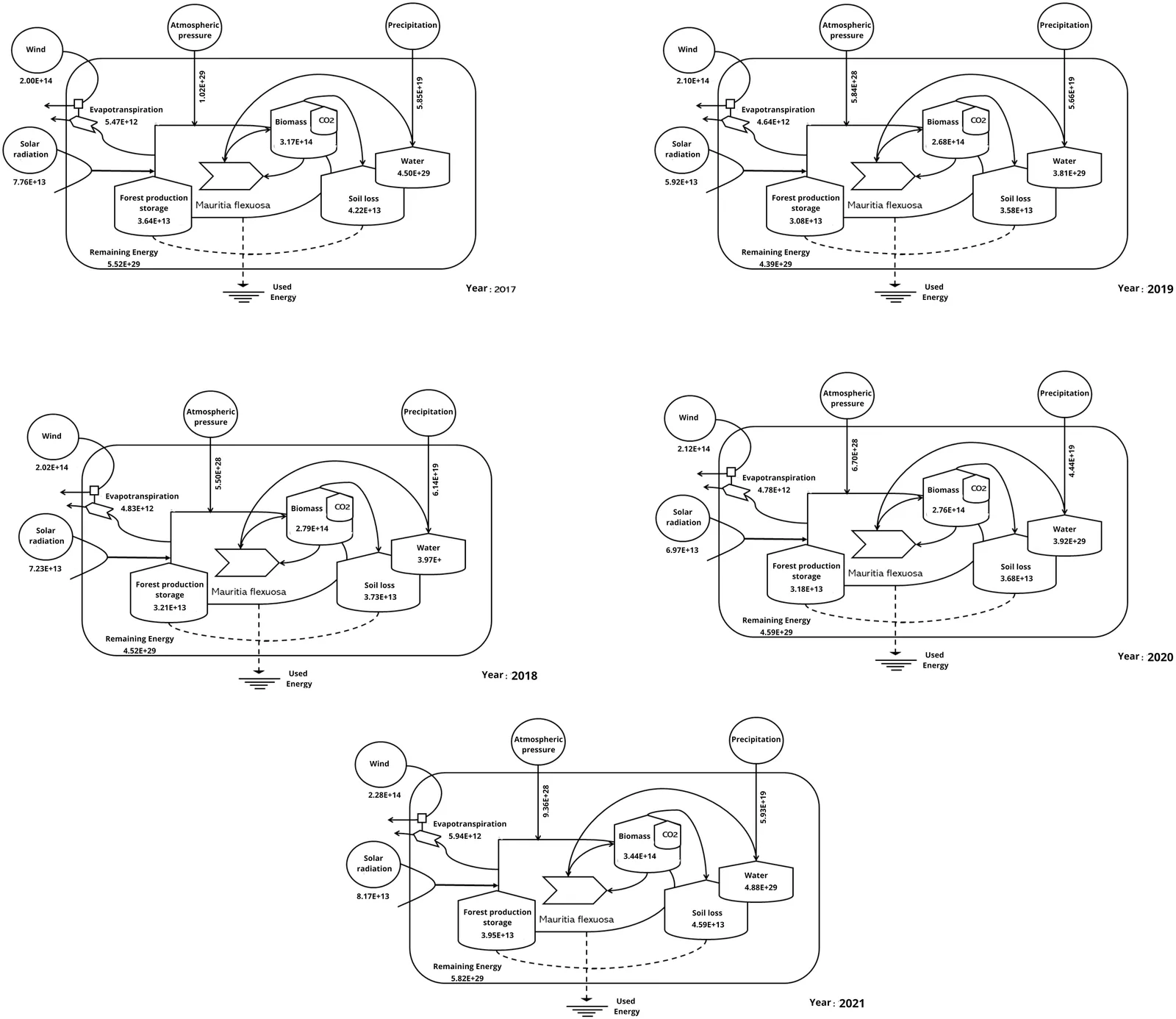
Emergy Flow Diagrams of the Cananguchales Ecosystem (*Mauritia flexuosa*) for Each Year Without Anthropogenic Intervention. Note. The figure shows the emergy flow diagrams for each of the analyzed years. Source: Own elaboration (2025).

From a comparative perspective, noticeable fluctuations are identified in the magnitude of certain energy flows—such as solar radiation (lowest in 2019 and highest in 2021) and atmospheric pressure (lowest in 2018 and highest in 2021)—possibly due to interannual climatic variations. Additionally, the progressive increase in forest storage and biomass, especially in 2021, suggests a positive system performance in terms of energy and carbon capture. Conversely, soil loss and evapotranspiration show oscillations with peaks at the beginning and end of the study period, which could reflect environmental pressures or changes in vegetation cover (Fig 3).

**Fig 4:**
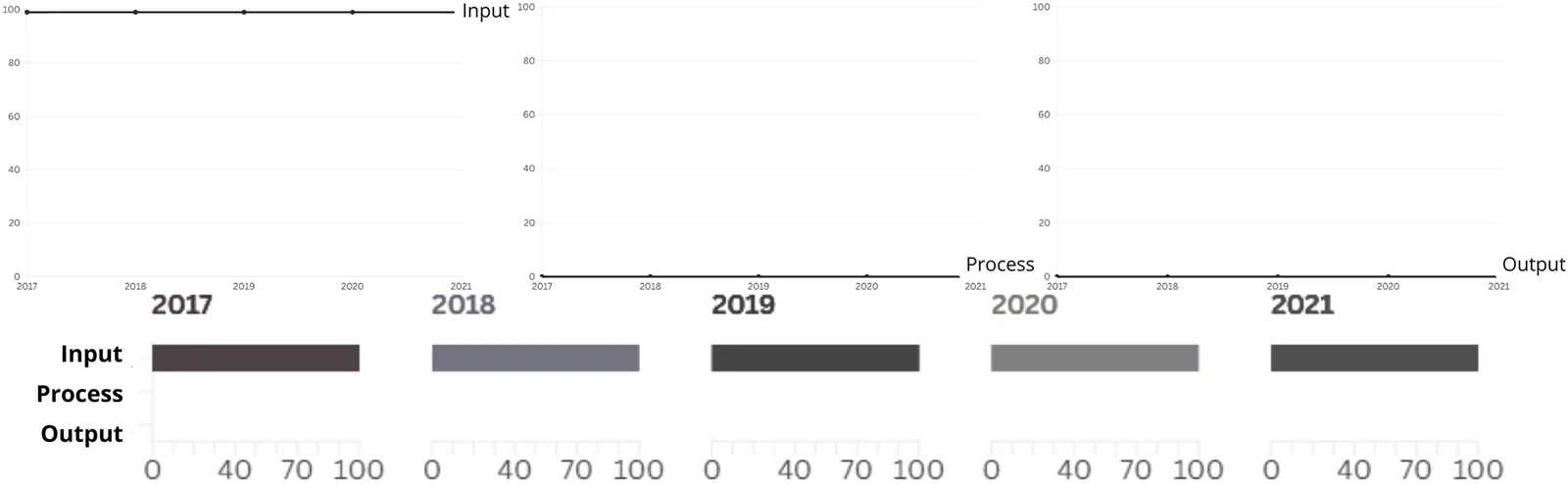
Inputs, Processes, and Outputs of the Cananguchales Ecosystem (*Mauritia flexuosa*) Without Anthropogenic Intervention. Note. The figure shows the biocentric valuation expressed in emergy-based US dollars for the most material variables of the undisturbed *Mauritia flexuosa* Cananguchales in the Amazonian foothills. The ecological dynamics are presented as Inputs (E), Processes (P), and Outputs (S). Resources or variables are classified as Renewable (R) and Non-renewable (N). Source: Own elaboration (2025).

Accordingly, Fig 4 clearly illustrates a predominance of energy input flows in the system, reaching levels close to 100% over the five-year period analyzed. This indicates a strong dependence on the biophysical environment for the functioning of the *Mauritia flexuosa* ecosystem. This condition is sustained by the consistent presence of renewable resources such as solar radiation, precipitation, and atmospheric pressure, which constitute the foundation of local ecological metabolism. In contrast, process and output components register considerably lower values, indicating that most available energy is absorbed or transformed internally but not necessarily exported or released significantly to other systems.

From a systemic perspective, this asymmetry suggests that *Mauritia flexuosa* acts as a node of energy accumulation and retention, as reflected in the progressive increase in biomass and forest storage over the five-year period. However, the low proportion of processed and released energy could imply limited efficiency in terms of ecosystemic performance, or alternatively, an adaptive strategy focused on resilience and internal sustainability. This pattern highlights the importance of maintaining and monitoring input flows, as any exogenous disturbance in these could significantly compromise the stability of the system.

Since calculating the variables in emergy units (Sej·year ^−^ ¹) is not sufficient for establishing a biocentric and sustainable value unit that offers a high degree of reliability regarding the ecological wealth of the provisioning ecosystem services in *Mauritia flexuosa* wetlands, it became necessary to transform the values from solar emergy joules (Sej) to emergy-based US dollars (Em$), as shown in Table 3. In simple terms, the conversion of solar emergy joules (sej) into emergy-based US dollars (Em$) follows the standard emergy-to-money ratio procedure: the total emergy (sej) that supports a reference economy — here, the regional economy of Caquetá — is divided by the annual monetary product of that economy (GDP, in US$), yielding an emergy-to-dollar ratio (sej/US$) for each year. Dividing the sej value of each ecosystem flow by that annual ratio returns its equivalent value in emergy-based dollars (Em$). This transformation allows for the valuation of energy inputs into the system in a unit comparable with financial information. The conversion to Em$ was performed by multiplying the usage ratio by the annual Gross Domestic Product (GDP) value in joules (J) for the system and the emergy of the prioritized variables of the provisioning ecosystem services in the *Mauritia flexuosa* wetlands, as expressed in the following equation:

**Table 3:** Biocentric valuation expressed in emergy-based US dollars (Em$) of the ecological wealth status of provisioning ecosystem services in *Mauritia flexuosa* wetlands.

Account/ Subaccount	2017	2018	2019	2020	2021	Accounting Dynamics	Dynamics	Ranking
	Value (US$)	Value (US$)	Value (US$)	Value (US$)	Value (US$)		Ecological	
**Assets**								
**Ecological Assets**								
***Ecological Inventory***								
**Radiation**	2.16E-03	3.98E-03	2.91E-03	2.48E-03	2.26E-03	Debit	E	R
**Wind Energy**	2.27E-06	4.55E-06	4.22E-06	3.09E-06	2.58E-06	Debit	E	R
**Rainfall**	5.35E-02	1.11E-01	9.14E-02	5.18E-02	5.37E-02	Debit	E	R
**Atmospheric Pressure**	1.16E + 09	1.24E + 09	1.17E + 09	9.75E + 08	1.06E + 09	Debit	E	R
**Evotranspiration**	5.88E-09	1.03E-08	8.81E-09	6.57E-09	6.34E-09	Debit	P	R
**Blue Water**	5.28E + 01	9.21E + 01	7.91E + 01	5.90E + 01	5.69E + 01	Debit	P	R
**Carbon Storage**	1.85E-17	3.23E-17	2.78E-17	2.07E-17	2.00E-17	Debit	P	R
**Forest Production Storage**	2.91E-08	5.06E-08	4.35E-08	3.24E-08	3.13E-08	Debit	P	R
**Total, Ecological Assets:**	**1.16E + 09**	**1.24E + 09**	**1.17E + 09**	**9.75E + 08**	**1.06E + 09**			
**Liabilities**								
**Liabilities Eco-friendly**								
**Soil loss**	1.59E-08	2.77E-08	2.38E-08	1.77E-08	1.71E-08	Credit	S	N
**Total, Liabilities Eco-friendly:**	**1.59E-08**	**2.77E-08**	**2.38E-08**	**1.77E-08**	**1.71E-08**			
**Remaining Energy**	1.16E + 09	1.24E + 09	1.17E + 09	9.75E + 08	1.06E + 09	Credit		R
**Total, Ecological Heritage:**	**1.16E + 09**	**1.24E + 09**	**1.17E + 09**	**9.75E + 08**	**1.06E + 09**			

*Note.* The table presents the biocentric valuation expressed in Em$ of the most materially significant variables in undisturbed *Mauritia flexuosa* Cananguchales in the Amazonian foothills. Ecological dynamics are shown as Inputs (E), Processes (P), and Outputs (S). Resources or variables are classified as Renewable (R) and Non-renewable (N).Source: Own elaboration (2025).


$$\begin{array}{c} {US\$}_{Sej}=\left(\frac{(\text{GPD})(\sum_{k=\text{1}}^{n}J_{K})}{\left(\overset{―}{x}{\text{US}\$.\text{year}}^{-\text{1}}\right)}\right).({Sej.year}^{-\text{1}}) \end{array}$$

Where:

${US\$}_{Sej}$= Emdollar Joules.

$\sum_{k=\text{1}}^{n}J_{K}$= Sum of Joules (J) of the system variables.

$\overset{―}{x}{\text{US}\$.\text{year}}^{-\text{1}}$= Annual average value of the dollar.

${Sej.year}^{-\text{1}}$= Emergy joule (sej) of the variable.

Thus, this valuation in monetary units enables the recognition of ecological wealth from the provisioning ecosystem services in *Mauritia flexuosa* wetlands using a unified structure aligned with financial variables used in organizations. In general terms, the emergy-based measurement and evaluation indices are proposed as tools to guide the interpretation and decision-making processes in biocountable disclosures regarding the ownership, control, use, exploitation, and circulation of ecological wealth in strategic provisioning ecosystems of the Amazonian foothills. This enables the integral and objective measurement of emergy efficiency (Sej) and non-financial information to support administrative management for sustainability and progress under differential and contextualized conditions of sequential ecological systems.

Within the biocounting framework, Table 3 reflects a patrimonial structure based on the recognition and valuation of ecological assets and liabilities in monetary terms, derived from energy flows converted into US dollars. Between 2017 and 2021, the ecological assets were primarily composed of atmospheric pressure, precipitation, and blue water—those with the highest economic values, indicating their high availability and functional relevance for the ecosystem. The accounting dynamics show that all assets are recorded as debits, which allows identification of the functional origin of each component based on its biophysical and economic logic.

On the other hand, ecological liabilities—represented by soil loss—are characterized as uncompensated energy outflows. These remain relatively low compared to asset values, which may indicate ecological system stability due to minimal human intervention. Finally, remnant energy is presented as ecological equity, reflecting the net energy balance as a sustainability indicator, which enables the establishment of a balance sheet aligned with accounting equations.

**Fig 5:**
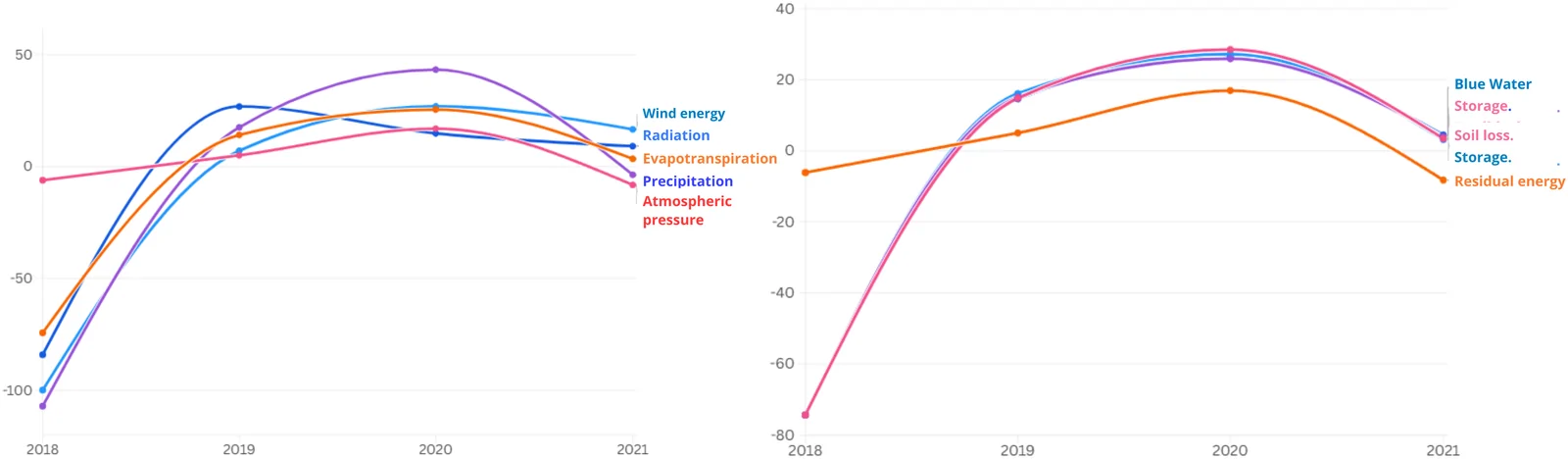
Behavior of the studied variables. Note. The figure shows the annual fluctuation of prioritized variables in the study, starting with the variation from 2017 to 2018 as Year 1. Source: Own elaboration (2025).

Fig 5 shows that the main energy input flows—such as wind energy, solar radiation, evapotranspiration, precipitation, and atmospheric pressure—exhibit oscillating behavior, with peaks in 2020 when precipitation showed the highest variation. All these variables confirm that Cananguchales are dynamic systems whose biophysical conditions are affected by external factors. Soil loss, in contrast, displayed lower intensity in its variation, which is a positive indicator for ecological conservation. Likewise, the behavior of remnant energy reflects the system’s net balance, with its decrease in 2021 potentially signaling a decline in energy efficiency or the system’s ability to sustain its internal metabolism.

Emergy analysis, as a biophysical valuation method based on thermodynamic principles, can be understood as a mechanism for recognizing ecological wealth within organizational reporting for decision-making purposes. In this sense, beyond assigning value to the biocountable processes of ecological assets and liabilities, it is necessary to represent such information through efficiency indices to evaluate the degree of sustainability in the management, control, use, exploitation, generation, and circulation of strategic ecological variables in territories—aimed at generating greater negentropy in both natural and anthropogenic productive processes.

Accordingly, the emergy indices used in this study—focused on the provisioning ecosystem services of *Mauritia flexuosa* wetlands in the Amazonian foothills—were the Ecological Loading Ratio (ELR), Emergy Investment Ratio (EIR), Emergy Yield Ratio (EYR), Renewability Index (%R), and Sustainable Emergy Index (ESI) [5,37,49]. The emergy analysis of prioritized variables revealed low levels of ecological stress, with an index of 1.72E-17, due to the system not being anthropogenically disturbed or dependent on market-imported or purchased emergy resources.

**Fig 6:**
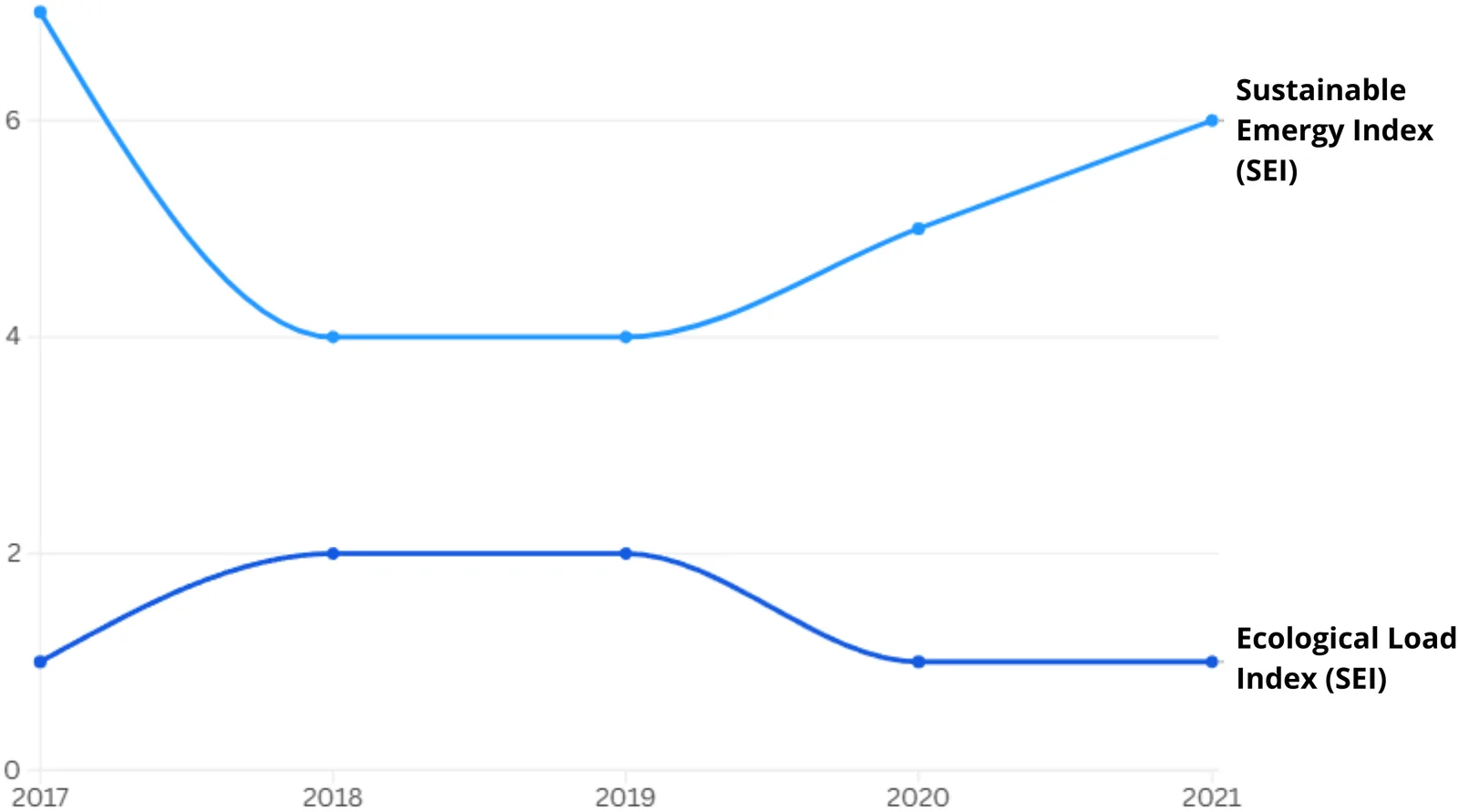
Behavior of the studied indices. Note. The figure shows the annual fluctuations of the Ecological Loading Ratio (ELR) and the Sustainable Emergy Index (SEI). Source: Own elaboration (2025).

Furthermore, Fig 6 shows the behavior of the Sustainable Emergy Index (SEI), which decreased between 2017 and 2018, stabilized in 2019, and began a steady increase, reaching a value in 2021 nearly equal to that of the initial year. This trend indicates a recovery in the system’s capacity to sustain its ecological functions through renewable sources, consistent with the observed increase in renewable wealth (R). The zero value in imported wealth (F), along with the constant values of the Emergy Yield Ratio (1) and the Renewability Index (100%) throughout the entire period, reinforce the idea of an autonomous system that relies exclusively on renewable inputs—thus strengthening its sustainable profile from an emergy perspective. This is particularly evident when the rise in remnant energy coincides with improvements in the SEI from 2020 onward.

In contrast, the Ecological Loading Ratio (ELR) shows the opposite behavior to the SEI, peaking between 2018 and 2019, and progressively decreasing until 2021. This suggests that pressure on natural resources gradually declined, due to greater use of renewable flows and the stabilization or reduction of ecological liabilities such as soil loss. From a systemic standpoint, the convergence of a decreasing ELR and an increasing SEI indicates that the analyzed ecosystem evolved toward a state of higher energy balance and sustainability. This type of analysis allows for assessing the ecological efficiency of the system beyond purely economic metrics.

To move beyond a purely descriptive reading of the five-year series, the annual values of the sustainability indices reported in Table 4 were characterised statistically. The Ecological Loading Ratio showed a mean of 1.82E-17 (SD = 3.40E-18; CV = 18.7%) and the Sustainable Emergy Index a mean of 5.67E + 16 (SD = 1.12E + 16; CV = 19.7%). Simple linear trends fitted against year were negligible in both cases (ELR: slope = 8.28E-20 year-1, R2 = 0.001; ESI: slope = −1.28E + 15 year-1, R2 = 0.033), indicating interannual fluctuation around a stable central value rather than a monotonic directional change. The Renewability Index remained above 99.99% and the Emergy Yield Ratio equal to 1 in every year, with no imported emergy (EIR = 0).

**Table 4:** Measuring indices of wetland sustainability with *Mauritia flexuosa* L.f. in the Amazonian foothills.

Description	Year 2017	Year 2018	Year 2019	Year 2020	Year 2021
NR: Non-renewable Wealth	5.71E + 08	5.03E + 08	4.83E + 08	4.98E + 08	6.20E + 08
F: Imported Wealth	0.00E + 00	0.00E + 00	0.00E + 00	0.00E + 00	0.00E + 00
R: Renewable Wealth	4.18E + 25	2.24E + 25	2.38E + 25	2.74E + 25	3.82E + 25
Index					
Ecological Loading Ratio (ELR)	**1.37E-17**	**2.2428E-17**	**2.0281E-17**	**1.8194E-17**	**1.6231E-17**
Sustainable Emergy Index	**7.32E + 16**	**4.46E + 16**	**4.93E + 16**	**5.50E + 16**	**6.16E + 16**
Investment Emergy Index	**0**	**0**	**0**	**0**	**0**
Emergy Performance Index	**1**	**1**	**1**	**1**	**1**
Renewability Index	**100**	**100**	**100**	**100**	**100**

Note. The table shows the calculated indicators for each year based on the classification of ecological wealth. The wealth aggregates used to compute the indices (NR: non-renewable wealth, F: imported wealth, R: renewable wealth) are expressed here in energy units (J·year^−^¹), taken directly from the flow-value columns of Table 2, whereas Table 2 also reports the same flows converted into emergy (sej·year^−^¹). Because each flow carries a different transformity, the ratios computed on the energy basis are not numerically identical to those computed on the emergy basis; the indices reported in this table are the energy-based values. Recalculating them on the emergy basis yields the same order of magnitude and the same interpretation (ELR ≈ 8 × 10^−^¹⁷, %R > 99.99%, EYR = 1, EIR = 0), so the classification of the Cananguchal as an almost entirely renewable, very low-load system is unaffected by the choice of basis. Source: Own elaboration (2025).

Because emergy assessments depend strongly on the transformity coefficients adopted, a sensitivity analysis was performed in which each transformity listed in Table 1 was varied by ±10% and ±20% around its literature value and the indices were recalculated. Under a ± 10% variation the ELR ranged between 1.49E-17 and 2.22E-17 and the ESI between 4.64E + 16 and 6.93E + 16; under a ± 20% variation the ranges widened to 1.21E-17–2.73E-17 and 3.78E + 16–8.51E + 16, respectively. The Renewability Index remained above 99.99% and the Emergy Yield Ratio equal to 1 in all scenarios, since the system receives no imported emergy. The absolute magnitude of the indices is therefore sensitive to the transformities adopted, whereas the qualitative conclusion — an almost entirely renewable, low-load system — is robust to this uncertainty.

When contrasted with values reported in the emergy literature, the indicators obtained for the Cananguchal are consistent with those of unmanaged wetland and forest systems. Emergy assessments of wetlands and low-intervention forest ecosystems, such as the subtropical wetland studied by Tilley & Brown [50] and the deltaic marshes evaluated by Martin [51], report renewability fractions close to 100% and ecological loading ratios well below unity, because these systems are sustained almost exclusively by environmental inputs and receive no purchased emergy. In contrast, agricultural and managed systems assessed under the same framework [39,41,49] report substantially higher loading ratios and lower renewability, reflecting their dependence on imported and non-renewable inputs. The renewability above 99% and the very low ELR obtained here therefore locate the Cananguchal at the low-pressure end of the reported range for emergy-assessed ecosystems. This comparison is deliberately framed in terms of ranges and orders of magnitude rather than point-by-point numerical matching. Emergy valuation expresses every flow in solar emergy joules through transformity coefficients, and the reliance on those conversion factors — together with the uncertainty affecting the calculation of several of them — is a recognised limitation of the method [37]: two studies of comparable ecosystems may report indices that differ by orders of magnitude simply because they adopted transformities from different sources, baselines or system boundaries. Direct numerical comparison of emjoule values across studies is therefore not warranted unless the transformity set and the emergy baseline are the same, whereas the relative position of a system (renewable-dominated and low-load versus imported-input-dependent and high-load) remains comparable and is the basis used here. A further constraint on the comparison should be made explicit: to the best of our knowledge, the emergy-based studies available for wetland and forest ecosystems have been conducted in temperate, subtropical or deltaic settings, and we identified no prior study applying emergy synthesis as a valuation method within the accounting sciences for the Colombian Amazon or, more generally, for humid tropical conditions. The comparison is therefore made against systems that differ in climatic regime, hydrological dynamics and biomass productivity from the Cananguchal, which is an additional reason to read it as an ordering of relative positions rather than as a numerical benchmark. Providing the first emergy-based accounting valuation for a humid tropical Amazonian wetland, and thereby a baseline against which future studies in comparable settings can be contrasted, is part of the contribution this study seeks to make.

Two limitations should be acknowledged, bearing in mind that the study is positioned within multidimensional accounting theory and that its purpose is the accounting recognition, measurement and disclosure of ecological wealth rather than statistical inference about ecosystem dynamics. First, with only five annual observations, formal significance testing and confidence-interval estimation for a trend (e.g., Mann-Kendall) have low statistical power and are consequently not reported; the coefficient of variation and the slope of a simple linear regression are presented instead as transparent descriptive measures of variability and directionality. Extending the monitoring series is recommended so that formal trend testing becomes feasible. Second, the results depend on transformity coefficients taken from the literature and derived for other geographical contexts; the sensitivity analysis reported above quantifies the effect of this uncertainty but does not eliminate it, and the derivation of locally calibrated transformities for Amazonian foothill wetlands remains an avenue for future research.

## Conclusions

Biocounting represents an evolution of traditional accounting by integrating the value of biophysical and ecological flows into the accounting framework, allowing for the quantification and recording of natural capital in both energetic and economic terms. It recognizes ecological assets and liabilities associated with ecosystem services. In this context, for *Mauritia flexuosa* Cananguchales, biocounting becomes a fundamental tool for measuring efficiency and performance within a sustainability framework using emergy indicators. It should be stressed that the emergy indicators themselves (ELR, ESI, EYR, EIR, %R) are established instruments and are not claimed as an innovation of this study. What distinguishes this approach from previous emergy-based ecosystem assessments, which report indicators as stand-alone ecological metrics, is that emergy-derived values are structured within a double-entry recognition framework (Table 3) designed for organisational and territorial sustainability disclosure, following an accounting logic grounded in multidimensional accounting theory rather than a purely ecological-modelling one, and therefore transferable to the recognition, measurement and disclosure of any type of wealth in any type of organisation. The contribution is also empirical and geographical: to the best of our knowledge, no previous study has applied emergy synthesis as a valuation method within the accounting sciences in the Colombian Amazon or under humid tropical conditions, so this work provides a first reference case for that setting.

The biocounting system developed in this research classified renewable energy flows—such as solar radiation, precipitation, atmospheric pressure, evapotranspiration, blue water, and both carbon and forest storage—as ecological assets. Meanwhile, soil loss was considered an ecological liability, representing an energy outflow from the system. The emergy synthesis shown in Table 2 revealed the energy flows and their transformities in sej/unit, with Table 3 presenting their monetary values, enabling decision-makers to interpret this information in conventional currency. Additionally, remnant energy was presented as the net energy balance available for system functionality. From this perspective, biocounting not only records ecological transactions but also facilitates the evaluation of ecological metabolism efficiency.

Despite interannual variability in some input flows (such as solar radiation and wind), the system maintained a pattern of internal energy stability, with processes like forest storage and biomass production acting as core elements for energy retention. The low level of outputs (e.g., soil loss) and the absence of imported wealth reinforce the strategic value of Cananguchales as natural energy sinks, essential for regional ecological resilience. The behavior of the ELR and SEI throughout the study period confirms that Cananguchales have improved their sustainability by reducing pressure on non-renewable resources and increasing the proportion of energy absorbed, transformed, and conserved. This reinforces their importance as a fundamental tool for guiding public policy and building biocounting frameworks that recognize the value of these ecosystems based on carrying capacity and resilience.

## Supporting information

S1 File Dry database Emergy Calculation. (XLSX)

S2 File Dry database Data compiled by IDEAM for the longitudinal assessment. (XLSX)

S3 File Dry database Glossary of Variables. (XLSX)
